# A study of head and shoulder posture and cervical muscle electromyographic characteristics in college students with chronic neck pain: A case–control study

**DOI:** 10.1097/MD.0000000000045789

**Published:** 2025-11-21

**Authors:** Yanqing Yan, Jifeng Dong, Taiping Li

**Affiliations:** a Guangdong University of Science and Technology, Dongguan, Guangdong, China.

**Keywords:** case–control study, chronic neck pain, electromyographic characteristics, flexion–relaxation responses, head and shoulder posture

## Abstract

Chronic neck pain (CNP) is a common musculoskeletal disorder among college students and is closely linked to poor head–shoulder posture and alterations in neuromuscular activity. Exploring postural and electromyographic (EMG) characteristics can provide essential insights for diagnosis and rehabilitation. This study aimed to comprehensively examine head–shoulder posture (including craniocervical angle, forward head posture [FHP], supine acromial distance, and rounded shoulder posture [RSP]) and cervical muscle function (strength and EMG characteristics) in college students with CNP to provide a reference for clinical diagnosis and rehabilitation. A total of 22 college students with CNP and 22 healthy students were recruited. Head–shoulder posture was evaluated using the craniocervical angle, supine acromial distance, FHP abnormality rate, and RSP abnormality rate. The isometric strength of the neck extensor muscles was measured at the neutral, natural anteversion, and maximum forward flexion positions. Surface EMG of the splenius capitis muscle and upper trapezius muscle was performed. Compared with their healthy peers, students with CNP had significantly smaller craniocervical angles and a greater incidence of abnormal FHP (*P* < .01). They had greater supine acromial distance and a greater incidence of a RSP (*P* < .01). Isometric muscle strength and EMG activity of the neck extensor muscles were significantly reduced in all positions (*P* < .01). During flexion–extension testing, the EMG flexion–relaxation response decreased, with CNP students showing higher flexion–relaxation ratio values (*P* < .01). College students with CNP show significant abnormalities in head–shoulder posture, decreased neck extensor strength, and altered EMG activity, including a diminished flexion–relaxation response. These findings highlight the critical role of postural and neuromuscular dysfunction in CNP, providing a valuable reference for clinical diagnosis and rehabilitation in the college student population.

## 1. Introduction

Chronic neck pain (CNP) is a prevalent musculoskeletal disease,^[1–6]^ particularly significant in the college student population,^[7–12]^ due to long periods of sedentary behavior, especially during study sessions and when using electronic devices. Poor head and shoulder posture is usually a pathogenic factor of CNP.^[13]^ Studies have shown that CNP patients in the university population often show abnormal craniocervical angles and forward head posture (FHP), which is considered to be a biomechanical risk factor for CNP.^[14–16]^ Additionally, electromyographic (EMG) studies have demonstrated that individuals with CNP exhibit increased activity in the neck muscles, particularly the splenius capitis and the superior trapezius. This enhanced muscle activation may lead to the occurrence and persistence of CNP.^[17,18]^ These findings suggest that the interaction between head–shoulder posture and neck muscle function may play an important role in the occurrence and development of CNP.

Several studies have examined the relationship between head–shoulder posture and neck muscle activity.^[13,19–22]^ Studies have shown that abnormal head posture, particularly head-forward posture, can lead to changes in the biomechanics of the cervical spine, which in turn increases the burden on the neck muscles and may cause muscle fatigue, sensory disorders, and increased pain.^[23]^ In addition, studies have found that FHP is closely related to increased activity of neck-stabilizing muscles, such as the superior trapezius and splenius capitis muscles.^[15,24]^ EMG analysis further demonstrated that CNP patients often showed changes in muscle activity patterns of the cervical flexor and extensor muscles^[6]^; these changes may lead to reduced movement efficiency^[25]^ and promote the occurrence of pain.^[26,27]^ These studies have jointly emphasized the important role of head–shoulder posture and neck muscle activity in CNP, providing a theoretical basis for further research.

This study aims to address this research gap by systematically examining the relationship between head–shoulder posture and the EMG characteristics of neck muscles in college students with CNP. By integrating postural and neuromuscular perspectives, this research aims to clarify how these factors jointly contribute to the development of CNP. This work enhances the theoretical understanding of mechanisms underlying CNP within the college student population and provides practical evidence for clinical practice, recommending posture assessment and EMG characteristics as reference points for diagnostic and rehabilitation strategies.

## 2. Methods

### 2.1. Participants

A case–control design was used in this study. At a significance level of 0.05 and a statistical power of 0.8, G*Power software calculated the recommended sample size as 30 participants (15 per group). Participants were recruited openly through campus announcements and email notifications, ensuring that all students meeting the inclusion criteria had an equal opportunity to participate. The research team conducted initial screenings, interviews, and physical examinations to ensure compliance with the inclusion and exclusion criteria. In this study, 44 college students aged 18 years or older were recruited between March 3 and April 5, 2020. In practice, we considered that significant individual variability may reduce the effect sizes; observational studies may encounter missing data or participant dropouts. Therefore, we ultimately included 44 patients (22 in the CNP group and 22 in the healthy control group) to ensure more representative and statistically robust results.

Written informed consent was obtained from all participants prior to enrollment. The consent process included a signed document outlining the study’s purpose, procedures, potential risks and benefits, confidentiality, and the voluntary nature of participation. No minors were included in the study, and no consent waiver was requested or granted. The experiments reported in the article were conducted, and the experimental protocol was approved (approval code: 2022020886) by the Ethics Committee of The Guangdong University of Science and Technology in accordance with the Declaration of Helsinki. All participants agreed to participate in the test and signed informed consent forms. The basic information is shown in Table 1.

**Table 1:** Basic information of patients with the CNP syndrome and healthy participants (mean ± SD).

Index	Patients (N = 22)	Healthy (N = 22)
Age (yr)	23.2 ± 1.07	23.6 ± 1.86
Height (cm)	169.0 ± 8.05	169.2 ± 8.01
Weight (kg)	60.3 ± 10.6	61.0 ± 10.9
Gender (male/female)	10/12	10/12

CNP = chronic neck pain.

### 2.2. Inclusion criteria for patients

Referring to the diagnostic criteria of neck muscle pain,^[28–30]^ patients with CNP should meet the following inclusion criteria:

1. recurrent neck pain, stiffness, and discomfort;
2. restriction of head and neck movement and local tenderness in the muscles at the back of the neck;
3. duration of pain > 3 months;
4. voluntary participation and signing of informed consent.

### 2.3. Inclusion criteria for healthy subjects

Healthy subjects should meet the following inclusion criteria:

1. no neck pain;
2. the same gender and major as those of the patients and similar to the patients in height, weight, and age.

### 2.4. Exclusion criteria for patients

Patients with any of the following were excluded:

1. accompanied by spinal cord and nerve root compression, hand numbness, dizziness, and other pathological phenomena;
2. with neck pain caused by a neck tumor, infection, and other reasons;
3. having a history of neck surgery, trauma, or congenital spinal abnormalities;
4. accompanied by severe cardiovascular and cerebrovascular disease, hypertension, diabetes, etc.

### 2.5. Test indicators and methods

#### 2.5.1. Craniocervical angle

Measuring instruments: joint angle ruler, tape, body posture assessment chart, and signature pen.

**Figure 1.**
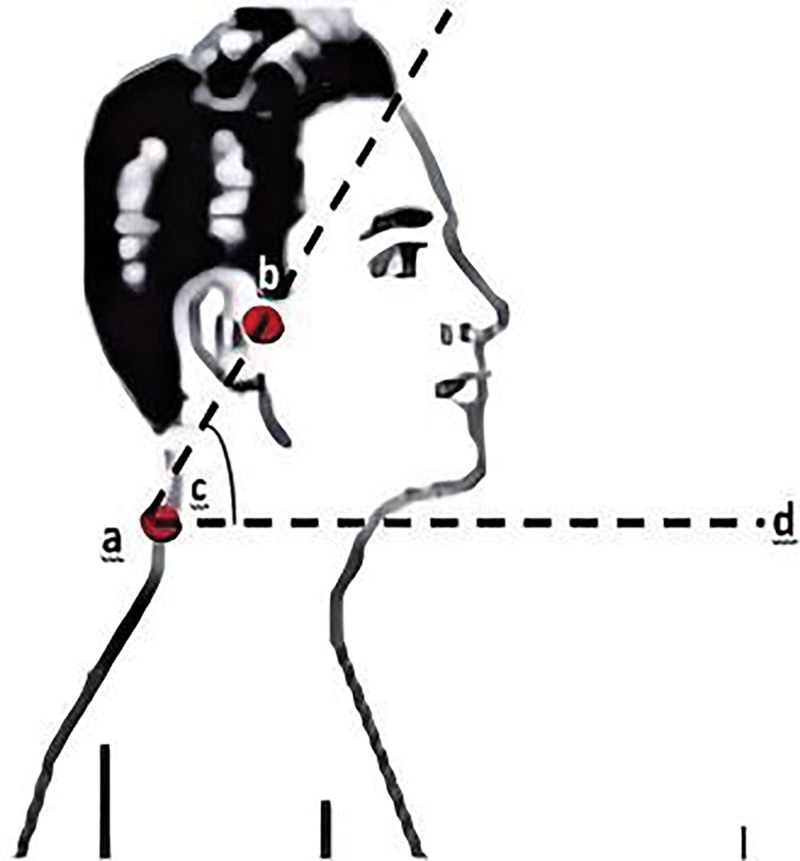
Craniocervical angle.

**Figure 2.**
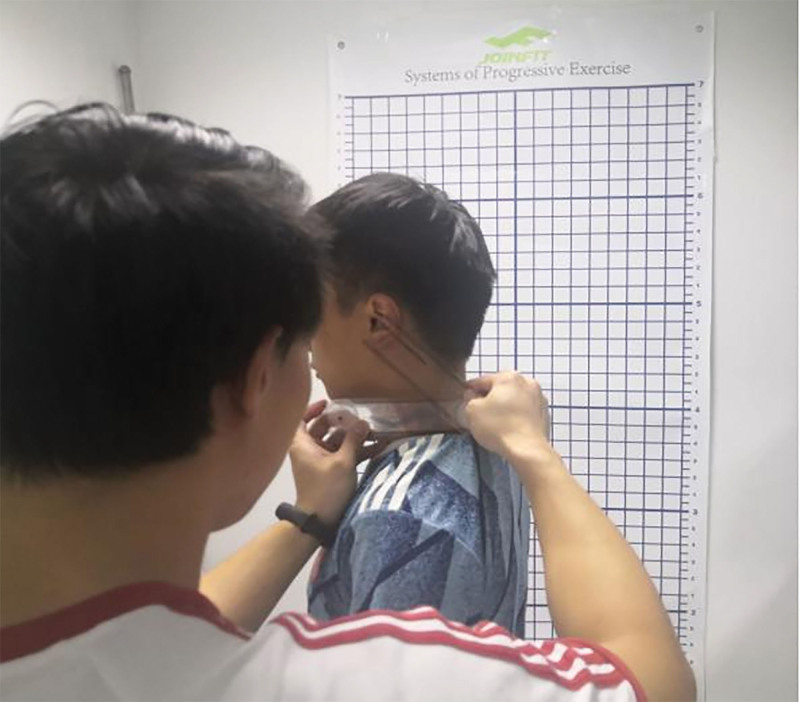
Measurement of craniocervical angle.

The FHP was determined by measuring the craniocervical angle, and a craniocervical angle of <48° was defined as an abnormal FHP.^[31,32]^ The craniocervical angle is the angle formed by the line between the tragus and the spinous process of the 7th cervical vertebra intersecting the horizontal line (as shown in Fig. 1); the smaller the craniocervical angle, the greater the degree of FHP. The participant was 1st asked to stand upright next to the posture assessment chart with eyes looking straight ahead, arms hanging naturally, and feet together. The horizontal line was positioned according to the horizontal line on the grid of the posture assessment form as a reference. Based on the height of the corner of each subject’s eye to the floor, a mark was then made on the wall as a reference point for keeping the eye at the horizontal plane. When the participant was in a natural forward head position (in the horizontal plane with the eyes looking flat to the front reference; the superior and inferior middle trapezius bundles were in a relaxed state), the spines of C7 were identified by palpation and marked with a small tape, and the value between the fixed and mobile arms at this point on the dial was read and recorded after measurement (as shown in Fig. 2); a total of 3 measurements were taken and the average value was taken.

#### 2.5.2. Supine acromial distance

**Figure 3.**
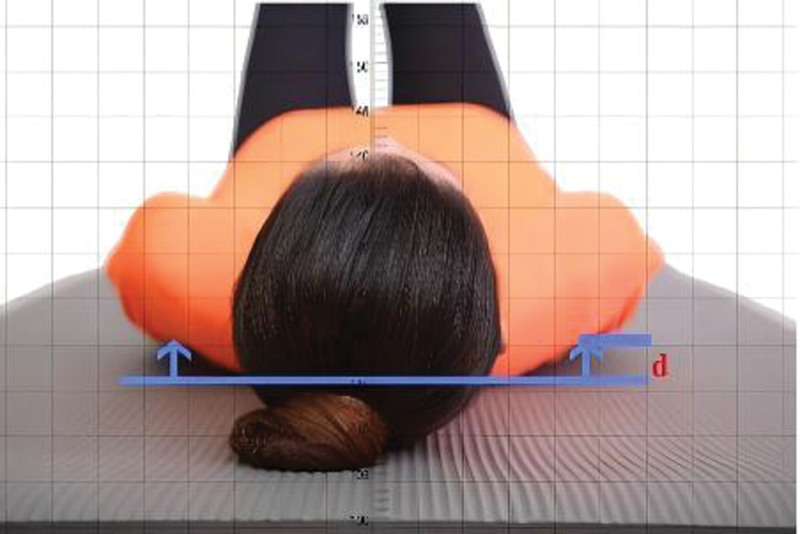
Measurement of supine acromial distance.

**Figure 4.**
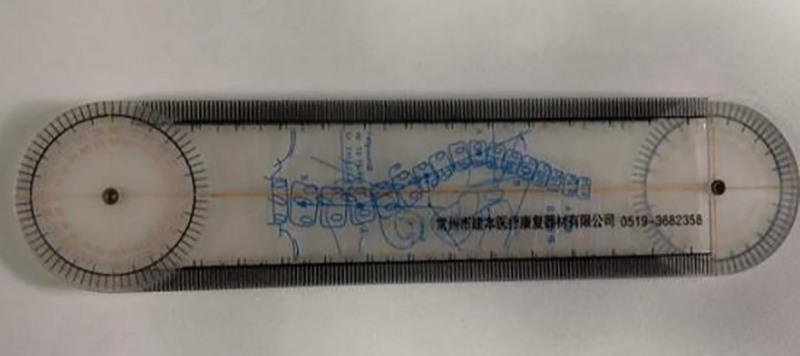
Joint angle rule.

The rounded shoulder posture (RSP) was determined by measuring the supine acromial distance, which was defined as abnormal when it was >2.6 cm.^[33]^ The participants lay supine in a stationary flat position (the supine position for measuring the acromial distance avoids measurement errors caused by shoulder rotation and scapular movement) on a yoga mat, and the RSP was measured in relation to the position of the scapula. When the shoulder is in a neutral position, measure the distance from the acromion to the surface of the yoga mat (as shown in Fig. 3) in centimeters using the joint angle ruler (as shown in Fig. 4) and take the average of 3 measurements.

#### 2.5.3. Maximum isometric strength of neck extensor muscles

##### 2.5.3.1. Instrumentation

**Figure 5.**
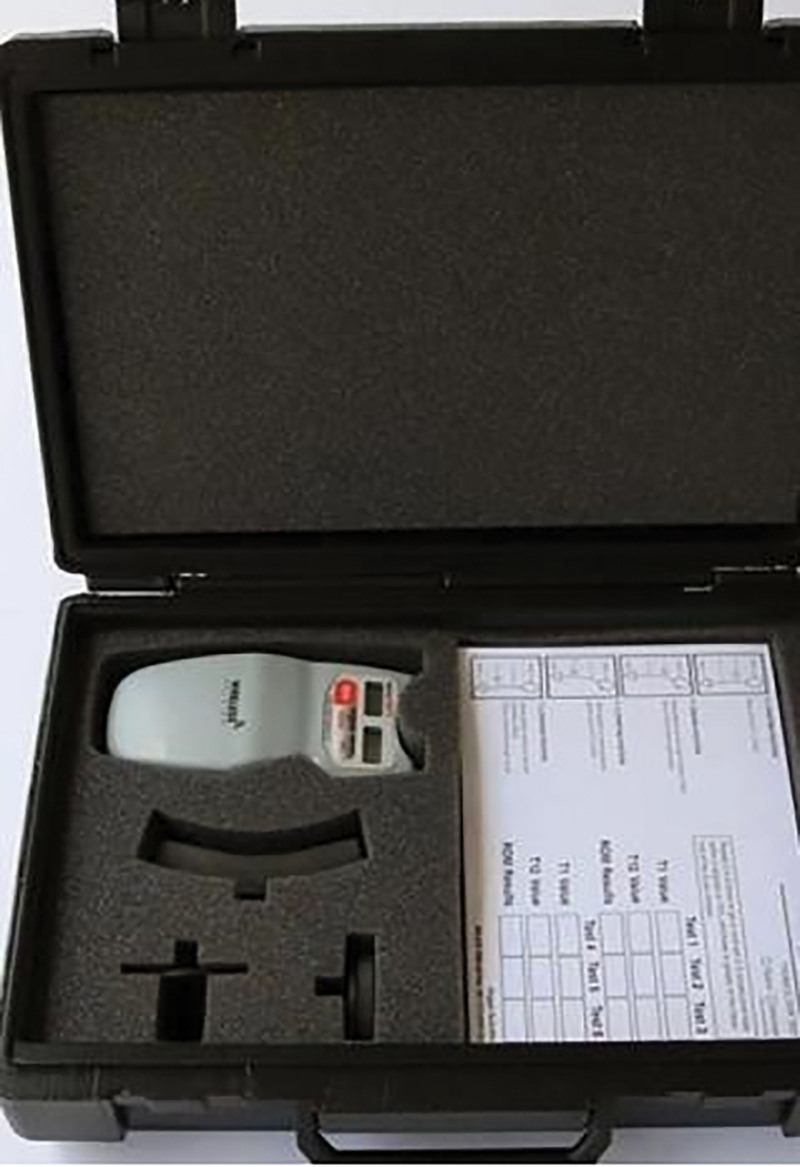
The wireless handheld digital muscle strength tester.

A wireless handheld digital muscle strength tester, as shown in Figure 5, was used.

##### 2.5.3.2. Procedure

In this study, kilogram-force was used as the unit of muscle strength. The maximum voluntary contraction of subjects’ neck extensor muscles was measured in a head neutral position, a natural anteversion position, and a maximum forward flexion position, respectively. After each test, the peak force and duration were displayed, and then the tester was reset.

#### 2.5.4. Static surface EMG of neck muscles

##### 2.5.4.1. Instrumentation

**Figure 6.**
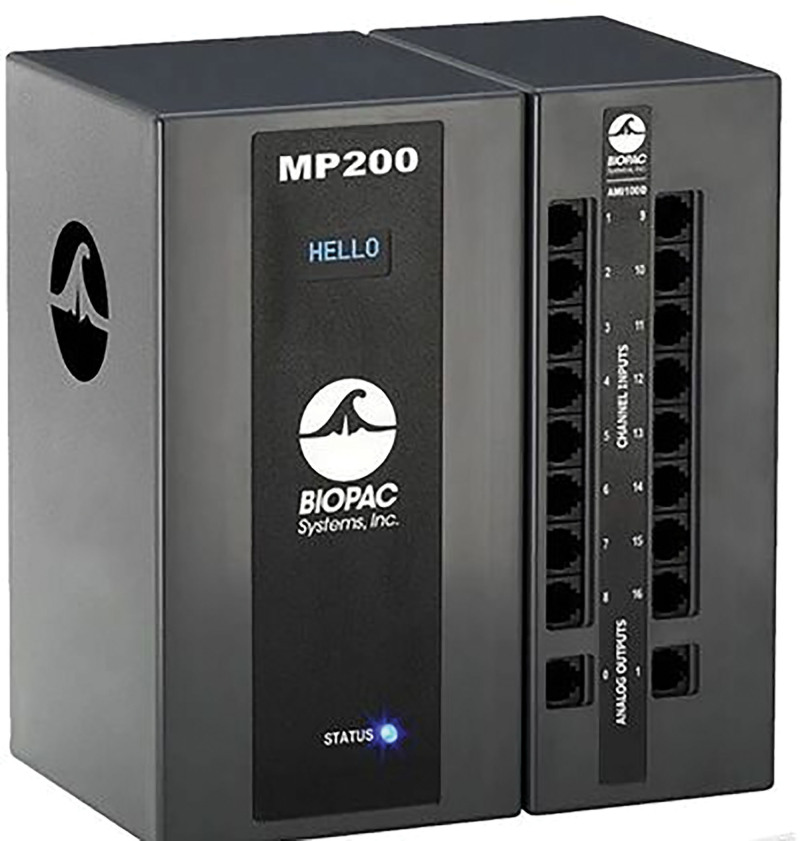
Surface electromyography tester.

A 16-channel physiograph BIOPAC MP200 was used to record the EMG signals in this study (as shown in Fig. 6). The EMG module was used to collect EMG signals. AcqKnowledge 4.2 (BIOPAC Systems, Inc., Goleta) was used to analyze EMG signals, and the sampling frequency was set to 1 kHz.

##### 2.5.4.2. Procedure

**Figure 7.**
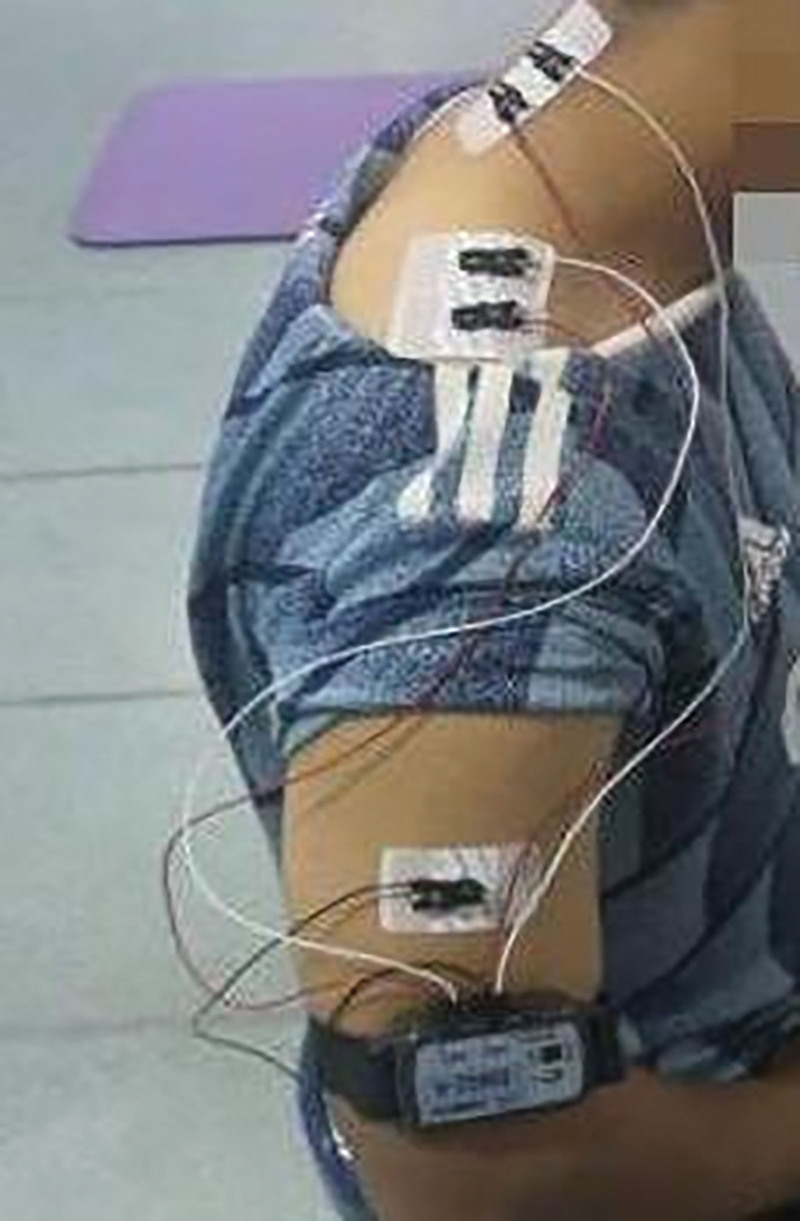
Electrode placement.

In static and dynamic surface EMG (sEMG) tests, the muscles to be tested are in the same position as the surface electrodes. During the test, the skin where the electrode is placed is cleaned with a 75% alcohol cotton ball to reduce skin resistance. The surface electrodes were placed in the superior trapezius muscle (the midpoint of the line connecting the spinous process of the 7th cervical vertebra and the acromion) and the splenius capitis muscle (1–2 cm away from the 4th cervical vertebra and the 5th cervical vertebra) of each subject, as shown in Figure 7. The centers of the positive and negative electrodes are 2 cm apart, and the diameter is 1 cm. After the surface electrode is pasted, it is reinforced with tape to prevent it from falling off during the experiment.

**Figure 8.**
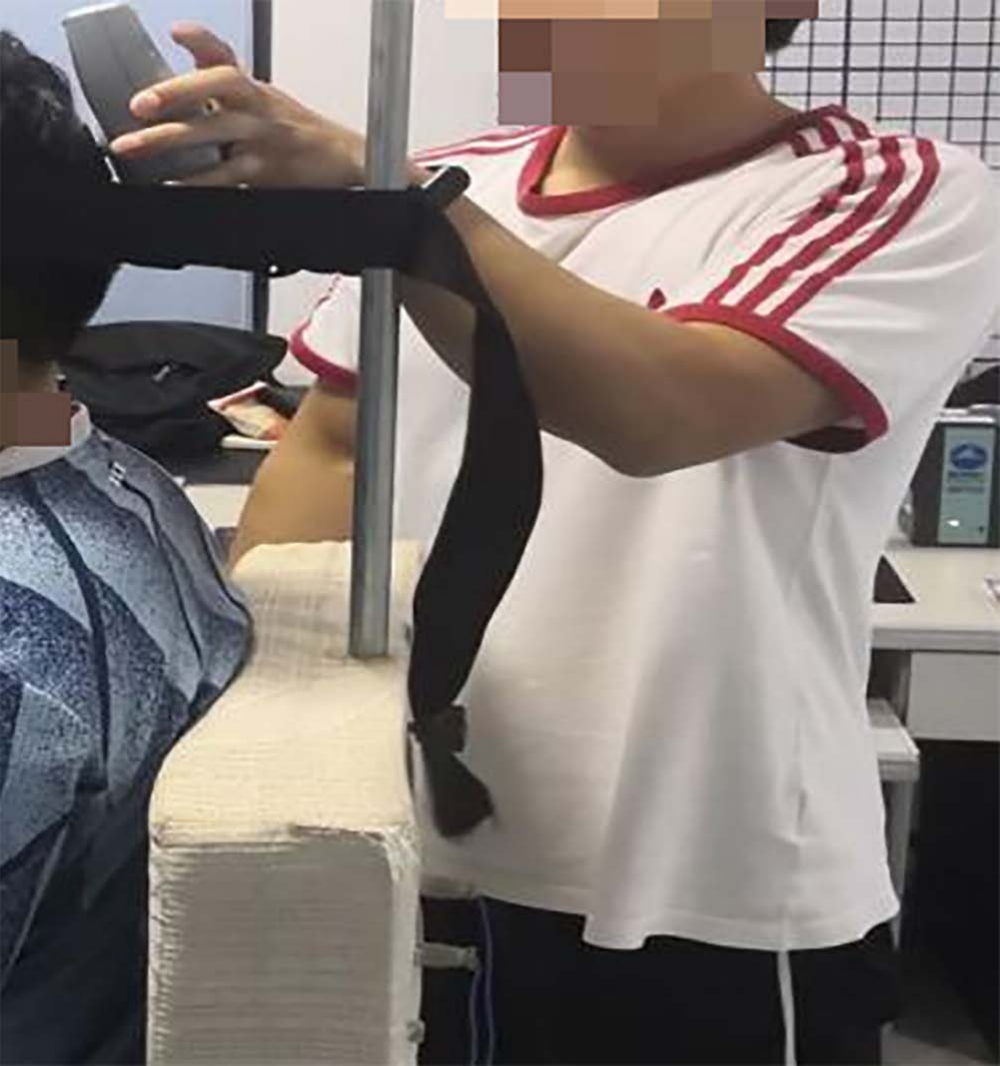
MVC test of neck extensor muscle in a head neutral position. MVC = maximum voluntary contraction.

**Figure 9.**
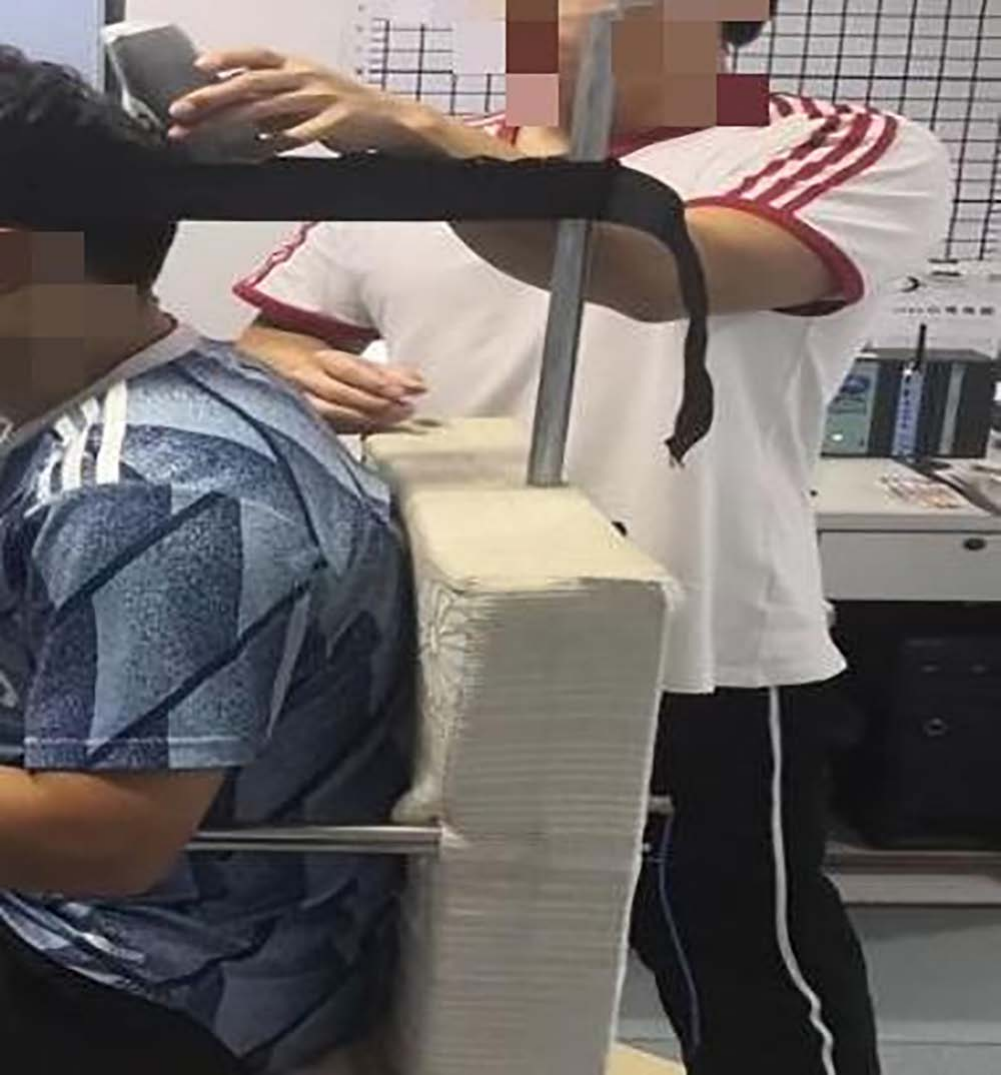
MVC test of neck extensor muscle in head natural anteversion position. MVC = maximum voluntary contraction.

**Figure 10.**
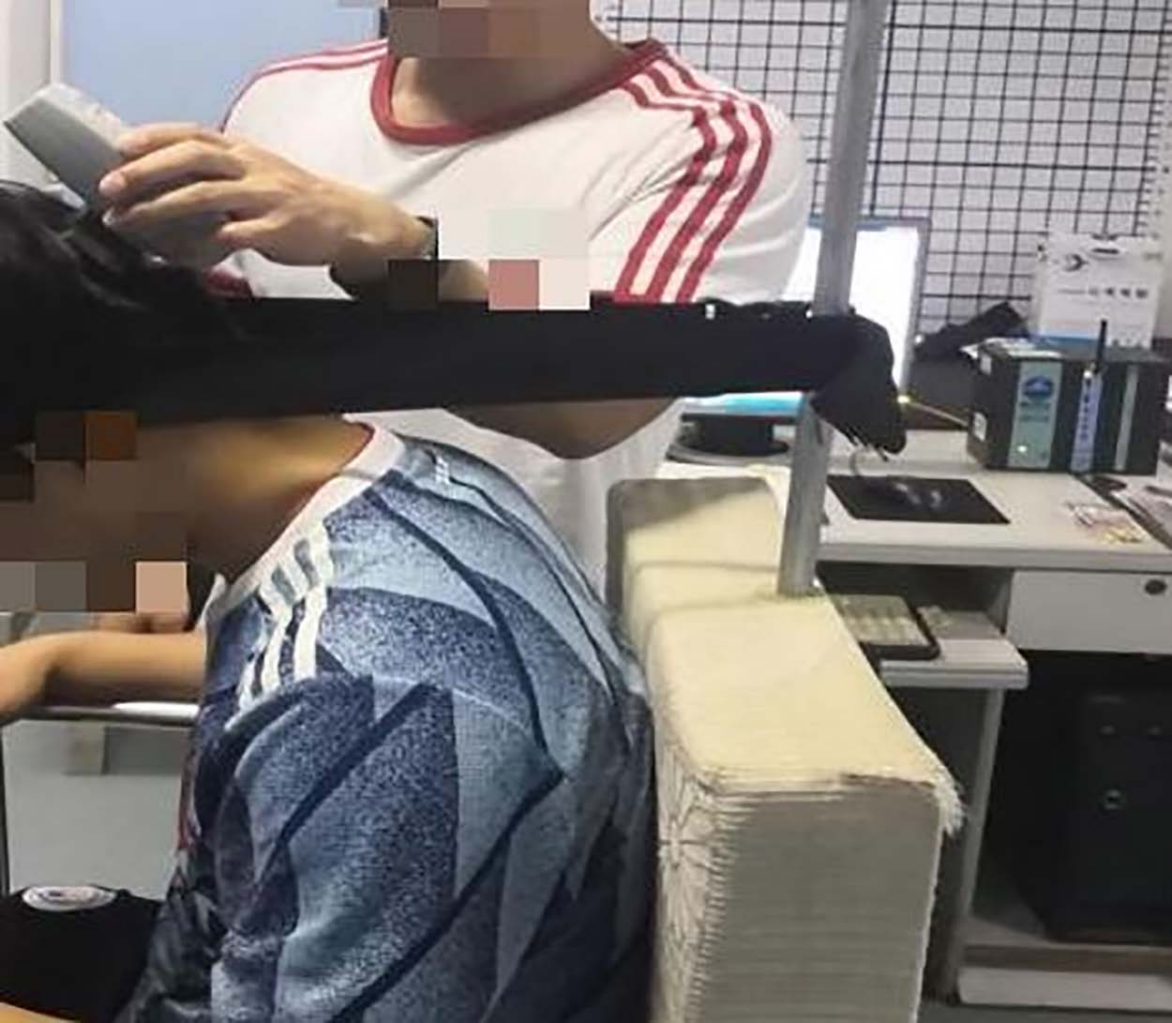
MVC test of neck extensor muscle in head maximum forward flexion position. MVC = maximum voluntary contraction.

**Figure 11.**
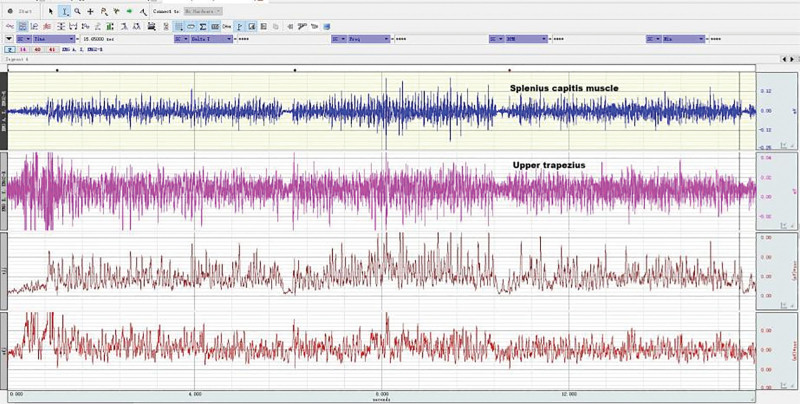
EMG in healthy subjects during the test of the maximum isometric strength of neck extensor muscles. EMG = electromyography.

**Figure 12.**
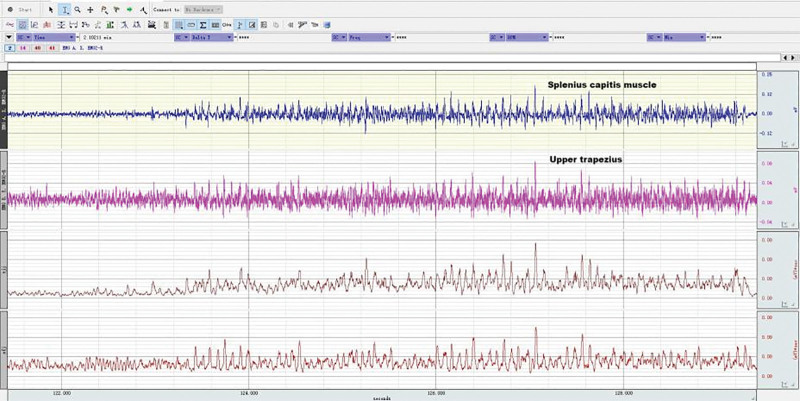
EMG in patients during the test of the maximum isometric strength of neck extensor muscles. EMG = electromyography.

The static sEMG tests of the splenius capitis muscles and the upper trapezius muscles were conducted at the same time as the maximum isometric strength of neck extensor muscles, as shown in Figures 8–10. EMG of each subject’s neck muscle in a head neutral position, natural anteversion position, and maximum forward flexion position was measured 3 times each. The EMG signals were analyzed. After the sEMG data were rectified, smoothed, filtered (10–500 Hz), and processed with standardization of amplitude, select 5 seconds of each group of data, a total of 3 groups, take the average value of the 3 groups to analyze average EMG (AEMG), integral EMG (iEMG) indicators by surface EMG software analysis and processing (as shown in Figs. 11 and 12).

Muscle iEMG contribution rate refers to the ratio of a specific muscle’s force development to all muscles’ force development in a certain stage of exercise, namely:


$$\begin{array}{l} Muscle\;iEMG\;contribution\;rate=\frac{iEMG\;of\;a\;specif\;ic\;muscle\;\;\;}{iEMG\;of\;all\;muscles}\times 100\% \end{array}$$

#### 2.5.5. Dynamic sEMG of neck muscles

##### 2.5.5.1. Procedure

**Figure 13.**
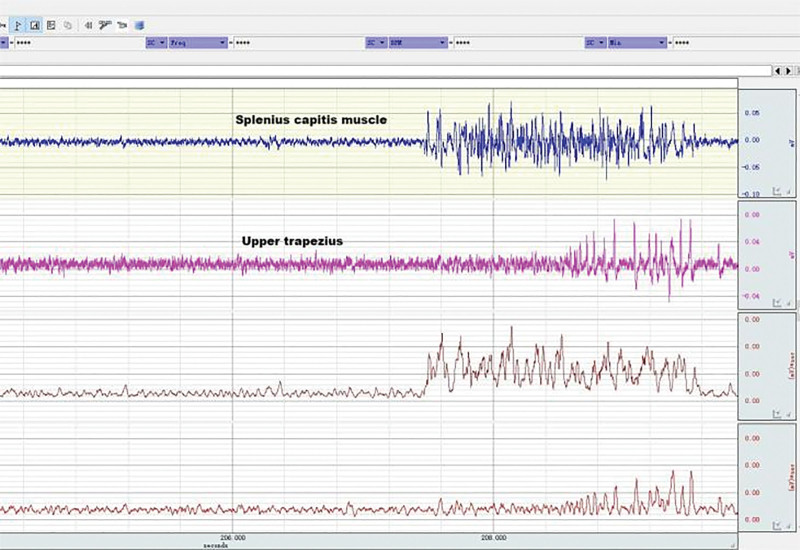
EMG of neck muscles in healthy subjects during neck flexion and extension. EMG = electromyography.

**Figure 14.**
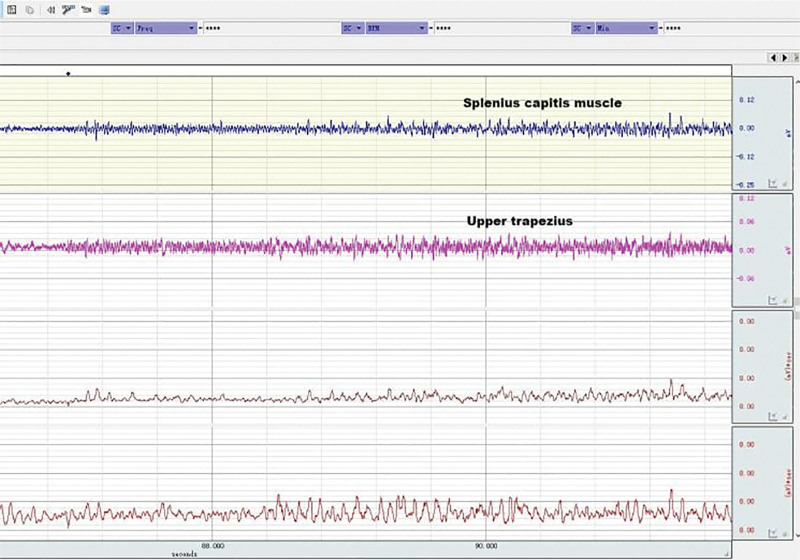
EMG of neck muscles in patient subjects during neck flexion and extension. EMG = electromyography.


During neck forward flexion and backward extension, the AEMG of each subject’s splenius capitis muscle and upper trapezius muscle was measured, and the AEMG ratios during flexion and extension were calculated. During the experiment, the electrodes were placed in the same manner as illustrated in Figures 3 to 5, 8. Each subject was required to sit in a chair with the feet shoulder-width apart. Then, the subject was instructed to slowly flex the neck forward to the maximum, holding the posture for 5 s before restoring the neck to the neutral position. The same action was repeated 5 times. Computer-generated sound feedback was used to prompt the subjects when to flex and extend, ensuring consistent speed of each flexion and extension. After the surface EMG data were rectified, smoothed, filtered (10–500 Hz), and processed with standardization of amplitude, the first 2 s and the last 2 s of each group of data in the 3 groups of data were selected. The raw sEMG signals were recorded, analyzed, and processed using the sEMG software, as shown in Figures 13 and 14. The flexion–relaxation ratio (FER) of the sEMG was then calculated using the following formula.

$$\begin{array}{l} FER\;=\frac{AEMG\;during\;flexion}{AEMG\;during\;extension} \end{array}$$

### 2.6. Experiment equipment

1. Surface EMG signals were collected using a BIOPAC MP200 16-channel physiological signal acquisition and analysis system (BIOPAC Systems Inc., Goleta).
2. Handheld dynamometer, Lafayette Manual Muscle Tester, Lafayette Instrument Company, USA.
3. Joint angle rule, ChngZhou Jianben Medical Co., Ltd.

### 2.7. Data processing

The experimental data were statistically analyzed using IBM SPSS Statistics (Version 24.0; IBM Corporation, Armonk) and expressed as the mean ± standard deviation. The data of patients and healthy subjects were compared and analyzed using the independent-samples *t* test. *P* < .05 was considered statistically significant. *P* < .01 was considered statistically significant. For categorical variables such as abnormal posture rates (e.g., FHP and RSP), comparisons were performed using the chi-square test.

## 3. Results

### 3.1. Forward head posture of patients and healthy participants

**Figure 15.**
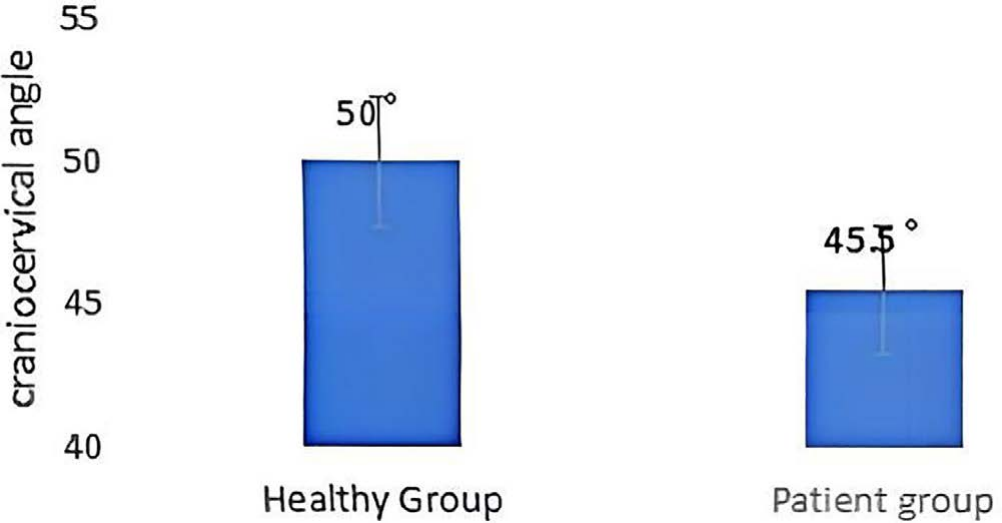
Forward head posture of patients and healthy participants.

As shown in Table 2 and Figure 15, the mean craniocervical angles of the patients with CNP and healthy participants were 45.5° and 50°, respectively, with highly significant differences between the 2 groups (*P* < .01); the abnormal rates of FHP were 100% and 27.2%, respectively, with highly significant differences (*P* < .01). The results indicated that CNP syndrome patients had smaller craniocervical angles than healthy participants and an abnormal forward head.

**Table 2:** Forward head posture of patients and healthy participants (mean ± SD).

Index	Patient group	Healthy group	*P* value
Craniocervical angle (°)	45.5 ± 1.63	50 ± 3.13	.001
The abnormal rate of forward head posture (%)	100	27.2	.001

### 3.2. Rounded shoulder posture of patients and healthy participants

**Figure 16.**
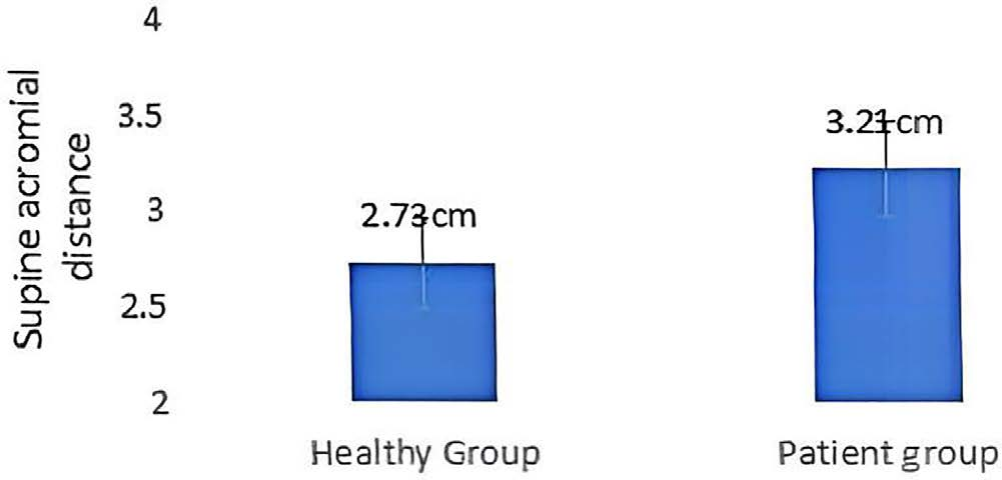
Rounded shoulder posture of patients and healthy participants.

As shown in Table 3 and Figure 16, the average supine shoulder peak distances of patients with CNP and healthy participants were 3.21 cm and 2.73 cm, respectively. The difference between the 2 groups was extremely significant (*P* < .01). The abnormal rate of the round shoulder was 100% and 72.7%, respectively. The difference between the 2 groups was statistically significant (*P* < .01). The results showed that the supine shoulder peak distance of patients with CNP syndrome was greater than that of healthy participants, and there was an RSP phenomenon in the patient group.

**Table 3:** Rounded shoulder posture of patients and healthy participants (mean ± SD).

Index	Patient group	Healthy group	*P* value
Supine acromial distance (cm)	3.21 ± 0.28	2.73 ± 0.29	.001
The abnormal rate of rounded shoulder posture (%)	100	72.7	.021

### 3.3. Isometric strength of neck extensor muscles in patients and healthy subjects

**Figure 17.**
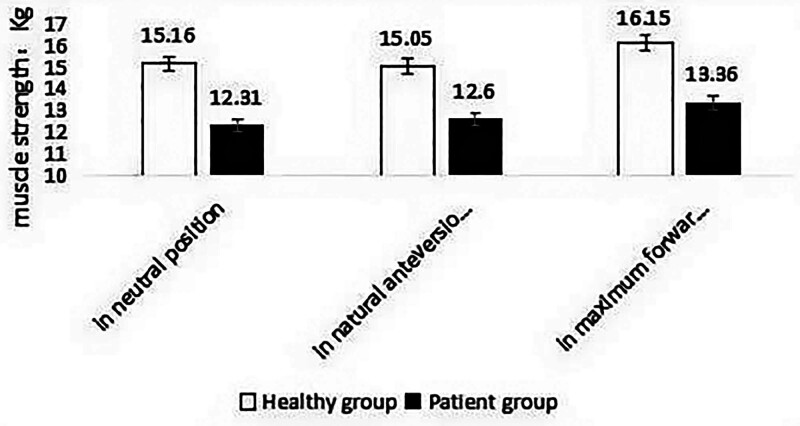
Isometric strength of neck extensor muscles in patients and healthy participants.

Table 4 and Figure 17 show the average isometric muscle strength of the neck extensors of patients and healthy subjects at 3 different head positions. In the neutral position of the head, the average value of the maximum isometric strength of the neck extensors of CNP patients and healthy subjects was 12.31 kg and 15.16 kg, respectively, and the ratio of patients to healthy subjects was 81%. The difference between the 2 groups was significant (*P* < .01). In the natural forward position of the head, the average values were 12.6 kg and 15.05 kg, respectively, and the ratio of patients to healthy subjects was 83%. The difference between the 2 groups was significant (*P* < .01). At the maximum flexion position of the head, the average values were 13.36 kg and 16.15 kg, respectively, and the ratio of patients to healthy people was 82 %. The difference between the 2 groups was significant (*P* < .01). The results showed that the overall level of isometric muscle strength of cervical extensors in CNP patients was lower than that in healthy subjects.

**Table 4:** Isometric strength of neck extensor muscles in patients and healthy participants (mean ± SD).

Head position	Patient group (unit: kg)	Healthy group (unit: kg)	Patient/healthy (%)	*P* value
In neutral position	12.31 ± 1.99	15.16 ± 2.50	81	.001
In natural anteversion position	12.60 ± 2.15	15.05 ± 2.36	83	.001
In maximum forward flexion position	13.36 ± 1.96	16.15 ± 2.66	82	.001

### 3.4. EMG in patients and healthy subjects during isometric contraction of neck muscles

#### 3.4.1. AEMG in patients and healthy subjects during isometric contraction of neck muscles

**Figure 18.**
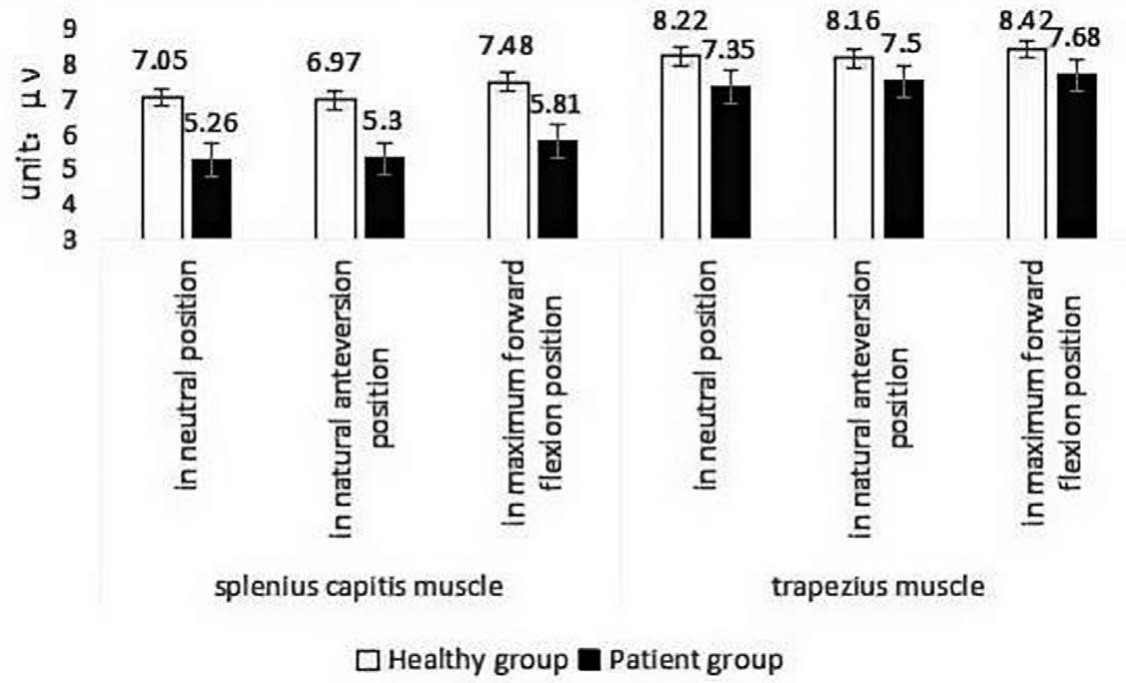
AEMG in patients and healthy participants during isometric contraction of neck muscles. AEMG = average EMG.

Table 5 and Figure 18 show the AEMG of patients and healthy subjects during isometric neck muscle contraction at 3 different head positions. In the head neutral position, AEMG of CNP patients and healthy subjects were 5.26 μv and 7.05 μv (splenius capitis muscle), respectively, and 7.35 μv and 8.22 μv (upper trapezius muscle) during isometric contraction of neck muscles. The ratio of AEMG to healthy subjects was 74 % and 89 %, respectively. The difference between the 2 groups was extremely significant (*P* < .01). At the natural anteversion position of the head, the average values were 5.3 μv and 6.97 μv (splenius capitis muscle) and 7.5 μv and 8.16 μv (upper trapezius muscle), respectively. The ratio of patients to healthy subjects was 76 % and 91 %, respectively. The difference between the 2 groups was extremely significant (*P* < .01). At the maximum flexion position of the head, the average values were 5.81 μv and 7.48 μv (splenius capitis muscle) and 7.68 μv and 8.42 μv (upper trapezius muscle), respectively. The ratio of patients to healthy subjects was 77 % and 91 %, respectively. The difference between the 2 groups was extremely significant (*P* < .01). The results showed that the overall level of AEMG in CNP patients was lower than that in healthy subjects during the maximum isometric contraction of neck muscles.

**Table 5:** AEMG in patients and healthy participants during isometric contraction of neck muscles (mean ± SD).

Muscle	Head position	Patient group (unit: μv)	Healthy group (unit: μv)	Patient/healthy (%)	*P* value
Splenius capitis muscle	In a neutral position	5.26 ± 1.04	7.05 ± 1.27	74	.001
	In natural anteversion position	5.30 ± 0.90	6.97 ± 0.92	76	.001
	In maximum forward flexion position	5.81 ± 0.99	7.48 ± 1.04	77	.001
Upper trapezius muscle	In neutral position	7.35 ± 0.81	8.22 ± 0.74	89	.001
	In natural anteversion position	7.5 ± 0.89	8.16 ± 0.89	91	.001
	In maximum forward flexion position	7.68 ± 0.91	8.42 ± 0.73	91	.001

AEMG = average EMG.

#### 3.4.2. iEMG in patients and healthy subjects during isometric contraction of neck muscles

**Figure 19.**
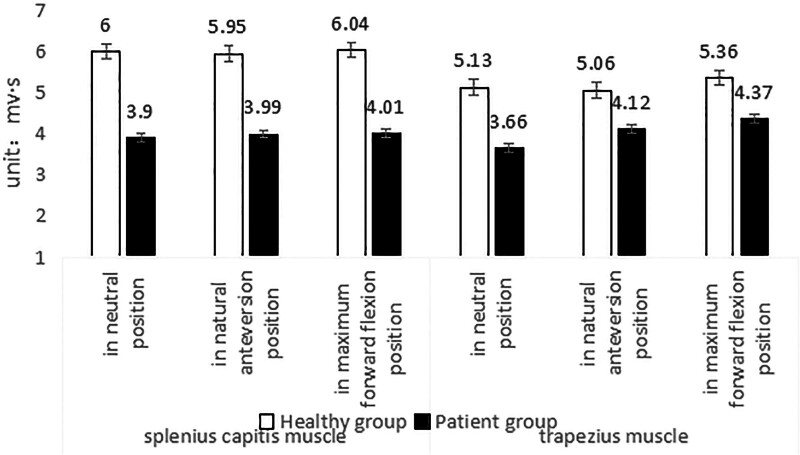
iEMG in patients and healthy participants during isometric contraction of neck muscles. iEMG = integral EMG.

Table 6 and Figure 19 show the mean values of iEMG during the maximum isometric contraction of neck muscles in CNP patients and healthy subjects at 3 different head positions. In the head neutral position, the average iEMG values of CNP patients and healthy subjects were 3.90 mv·s and 6.00 mv·s (splenius capitis muscle), 3.66 mv·s and 5.13 mv·s (upper trapezius muscle), respectively. The iEMG ratios of patients and healthy subjects were 65 % and 71 %, respectively. The difference between the 2 groups was extremely significant (*P* < .01). In the natural forward position of the head, the average values were 3.99 mv·s and 5.95 mv·s (splenius capitis muscle), 4.12 mv·s and 5.06 mv·s (upper trapezius muscle), respectively. The iEMG ratios of patients and healthy subjects were 67% and 81%, respectively. The difference between the 2 groups was extremely significant (*P* < .01). At the maximum flexion position of the head, the average values were 4.01 mv·s and 6.04 mv·s (splenius capitis muscle) and 4.37 mv·s and 5.36 mv·s (upper trapezius muscle), respectively. The iEMG ratios of patients and healthy subjects were 66% and 81%, respectively. The difference between the 2 groups was extremely significant (*P* < .01). The results showed that the overall level of iEMG in patients with CNP syndrome was lower than that in healthy subjects during the maximum isometric contraction of neck muscles.

**Table 6:** iEMG in patients and healthy participants during isometric contraction of neck muscles (mean ± SD).

Muscle	Head position	Patient group (unit: mv·s)	Healthy group (unit: mv·s)	Patient/healthy (%)	*P* value
Splenius capitis muscle	In a neutral position	3.90 ± 0.96	6.00 ± 1.32	65	.001
	In natural anteversion position	3.99 ± 1.22	5.95 ± 1.44	67	.001
	In maximum forward flexion position	4.01 ± 0.94	6.04 ± 1.38	66	.001
Upper trapezius muscle	In neutral position	3.66 ± 0.86	5.13 ± 0.77	71	.001
	In natural anteversion position	4.12 ± 1.27	5.06 ± 0.76	81	0.002
	In maximum forward flexion position	4.37 ± 0.90	5.36 ± 1.19	65	0.004

iEMG = integral EMG.

Table 7 shows the iEMG contribution rate of CNP patients and healthy subjects during the maximum isometric contraction of neck muscles at 3 different head positions. In the head neutral position, the contribution rate of iEMG during the maximum isometric contraction of cervical muscles in CNP patients and healthy subjects was 51.5% and 53.9% (splenius capitis), and 48.4% and 45.3% (trapezius superior), respectively. In the natural forward position of the head, the incidence was 49.1% and 54.0% (splenius capitis) and 50.8% and 45.9% (superior trapezius), respectively. At the maximum flexion position of the head, the incidence rates were 47.7% and 52.6% (splenius capitis) and 52.1% and 47.3% (trapezius superior), respectively. The results showed that the iEMG of the splenius capitis in CNP patients decreased significantly, while the iEMG of the superior trapezius decreased relatively small. Therefore, the muscle strength of the splenius capitis in CNP patients decreased more than that in healthy people, while the muscle strength of the superior trapezius decreased relatively small.

**Table 7:** iEMG contribution rates in patients and healthy participants during isometric contraction of neck muscles.

Head position	Muscle	Patient group (%)	Healthy group (%)
In neutral position	Splenius capitis muscle	51.5	53.9
	Upper trapezius muscle	48.4	45.3
	Total	100	100
In natural anteversion position	Splenius capitis muscle	49.1	54.0
	Upper trapezius muscle	50.8	45.9
	Total	100	100
In maximum forward flexion position	Splenius capitis muscle	47.7	52.6
	Upper trapezius muscle	52.1	47.3
	Total	100	100

iEMG = integral EMG.

### 3.5. Neck muscle EMG in patients and healthy subjects during head and neck flexion and extension

**Figure 20.**
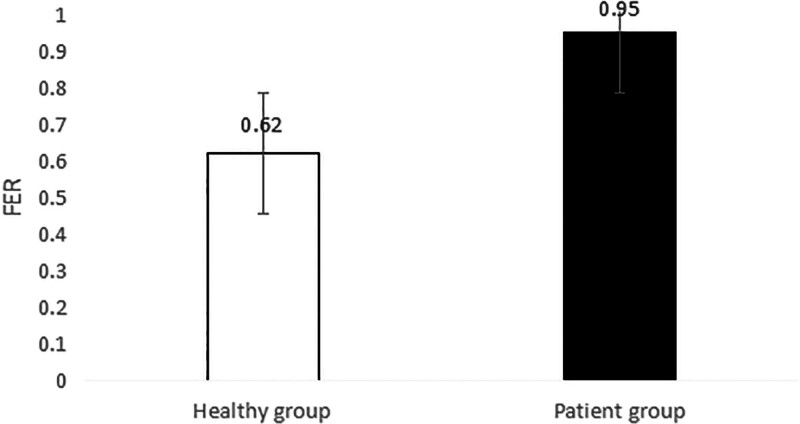
AEMG of neck muscles in patients and healthy participants during head and neck flexion and extension. AEMG = average EMG.

As shown in Table 8, during forward flexion, the average values of AEMG of splenius capitis muscles and upper trapezius muscles in patients with CNP added up to 62.99 μv, and those in healthy subjects added up to 55.79 μv. There was a very significant difference (*P* < .01). During backward extension, the average values of AEMG in patients with CNP and healthy subjects added up to 64.75 μv and 79.22 μv, respectively. There was a very significant difference (*P* < .01). As shown in Figure 20, the FERs of neck muscles in the 2 groups were respectively 0.95 and 0.62 on average. There was a very significant difference (*P* < .01). The higher the FER of sEMG, the worse the flexion–relaxation effect of neck muscles, indicating that patients with CNP syndrome were weaker than healthy subjects in terms of flexion relaxation of neck muscles, and there were painful changes in patients’ neck muscle function, showing the symptom of lack of flexion–relaxation response and insufficient active activity function.

**Table 8:** AEMG of neck muscles in patients and healthy participants during head and neck flexion and extension (mean ± SD)

Head and neck movement	Index	Patient group (unit: μv)	Healthy group (unit: μv)	*P* value
During forward flexion	AEMG of splenius capitis muscles	28.47 ± 6.82	25.13 ± 3.56	.002
	AEMG of upper trapezius muscles	34.52 ± 7.95	30.66 ± 4.66	.005
	Total	62.99 ± 14.77	55.79 ± 8.22	.007
During backward extension	AEMG of splenius capitis muscles	29.84 ± 6.09	40.06 ± 3.47	.001
	AEMG of upper trapezius muscles	34.91 ± 6.06	39.16 ± 4.62	.001
	Total	64.75 ± 12.15	79.22 ± 8.09	.001
	FER	0.95 ± 0.09	0.62 ± 0.08	.001

AEMG = average EMG, FER = flexion–relaxation ratio.

## 4. Discussion

### 4.1. Characteristics of head and shoulder posture in college students with CNP

According to the study results, the craniocervical angle in patients with CNP was significantly smaller than in the healthy group, indicating potential postural adaptation changes in patients with CNP. Normally, the craniocervical angle is approximately 50°^[31]^; it is considered to be the normal physiological range for maintaining good head and neck function. However, the reduced craniocervical angle in patients with CNP may be due to compensatory postural changes caused by long-term neck loading, muscle fatigue, and pain. Studies have shown that long-term poor neck posture may aggravate the load on the cervical spine and its surrounding soft tissues, thus aggravating the malignant cycle of muscle fatigue and pain.^[8,34]^

Further, the 100% FHP abnormality seen in CNP patients suggests that this type of postural abnormality is prevalent in CNP patients. FHP is usually closely associated with overactivity and imbalance of the neck muscles, especially the sustained overstrain of the cervical extensor muscle groups. As a common postural abnormality, FHP not only increases the load on the cervical spine in forward flexion but also may lead to dysfunction of the upper back, shoulders and lower limbs, which may aggravate neck pain.^[35]^ Thus, abnormalities in FHP may play a malignant circle in patients with CNP, allowing pain to continue and worsening the patient’s postural problems.^[36]^

In terms of shoulder posture, the supine scapular distance in CNP patients was significantly greater than that in the healthy group, and the abnormality rate of RSP was also significantly higher than in the healthy group. These results indicate that patients with CNP exhibit not only abnormalities in head posture but also changes in shoulder posture, which should not be overlooked. Round shoulder posture is usually associated with excessive tension of shoulder muscles (especially levator scapulae and trapezius) and abnormal alignment of shoulder joints.^[37,38]^ Long-term neck pain often leads to muscle imbalances in the shoulder, which can lead to changes in the position of the shoulder.^[39–42]^ The scapula is forwardly tilted, leading to a typical rounded shoulder.

The increased scapular distance in patients with CNP may be due to tension in the shoulder girdle muscles and dysfunction of the scapular fixation muscle groups (e.g., trapezius, levator scapulae, etc). The abnormal shoulder posture not only affects the function of the shoulder but may also negatively impact the load on the neck and back muscles, which in turn can aggravate neck pain.^[43,44]^ Thus, the phenomenon of rounded shoulders and increased scapular distance may reflect adaptive changes in the muscles and joints of the shoulder in patients with CNP, which may interact with dysfunction in the neck to exacerbate pain.

Postural abnormalities, especially changes in head and shoulder posture, may be one of the important causes of exacerbation of CNP symptoms. In this study, we observed that patients with CNP commonly exhibited postural problems, including a decreased craniocervical angle, abnormal anterior head posture, and increased scapular distance. These abnormalities may be attributed to muscle imbalance, muscle fatigue, and compensatory postural adjustments. Research has shown that changes in posture are often strongly associated with muscle and soft tissue overload, increased pain, and dysfunction. In patients with CNP, poor head and shoulder posture may lead to persistent muscle tension, which in turn worsens pain and makes treatment more difficult.

Therefore, improving abnormal head and shoulder posture may be an important part of CNP treatment. In clinical practice, corrective exercises targeting neck and shoulder posture, enhancing the strength and balance of neck and shoulder muscles, and improving muscle flexibility may help to reduce pain, restore normal posture, and reduce the recurrence of pain.

### 4.2. Characteristics of neck muscle strength in college students with CNP

Muscle strength is widely used in biomechanical research, and the condition of rehabilitation can be evaluated and analyzed by muscle strength.^[45]^ The energy of human movement comes from muscle strength, and the size of muscle strength can quantitatively evaluate the functional status of movement. Therefore, the study of muscle strength is of great significance in evaluating motor function and biomechanical analysis. Exercise therapy for the neck, such as traditional exercise therapy, Baduanjin, yoga, or more recent approaches like 3-dimensional static resistance training, isokinetic exercise, can enhance neck muscle strength, relieve spasms, improve blood circulation, and accelerate inflammation regression.^[46]^ It can improve the neck muscle strength and movement coordination of college students with CNP, restore the mechanical properties of the neck muscle group, delay fatigue, and achieve good results in maintaining the stability of the neck.^[47]^

The cause of CNP is related to neck muscle factors. Using the soft tissue tension test system, it is concluded that the neck muscle tension of patients with CNP increases, while their endurance decreases, which is closely related to changes in physical signs and neck function.^[36]^ Therefore, we should focus on the influence of neck muscle strength and endurance on neck biomechanics, which is the key to preventing neck pain and CNP. The instability of the neck is the cause of CNP pain. Strength training for the core muscles of the neck and body can reduce the pain and incidence of CNP and increase the flexibility and stability of the joints. Usually, the neck muscles relax and contract, maintaining the mechanical balance of the neck. Multiple muscles in the neck work together to coordinate and contract. These muscles exhibit characteristics of weak endurance, limited muscle strength, easy imbalance, and poor coordination. The main reason for the static and dynamic imbalance of the neck is the weakening of the flexion and extension function of the neck muscles. The biomechanical imbalance of muscles, ligaments, tendons, and joint capsules surrounding the neck is demonstrated.^[3]^

In daily life, repeated head-down work is a common cause of chronic CNP. The neck lacks a standard mode of action, the self-stabilizing mechanism is disordered, the physiological morphology of the cervical spine changes, and the intervertebral foramen narrows. The cause is that life and work are not based on biomechanics. The neck is the most flexible human joint, but its structure is also the most unstable. The neck relies on muscles, bones, and ligaments to maintain stability and execute complex, 3-dimensional motions that are both complete and changeable. When it is CNP, these action modes cannot be completed well, resulting in long-term compensatory contraction and eventually leading to CNP pain.^[48]^

In this experiment, the maximum isometric contraction was used to test the strength of the cervical extensor muscle, as its primary function is to maintain the movement and stability of the cervical spine (endogenous stability). This study found that the maximum isometric muscle strength of the splenius capitis and trapezius of college students with CNP was significantly lower than that of healthy college students. There was a significant difference in statistics (*P* < .01), indicating that the cervical extensor muscle strength of college students with CNP was relatively weak. There were significant differences in muscle strength between the 2 groups in the head neutral position, the head natural forward position, and the head maximum flexion position (*P* < .01). The study reveals that the cervical extensor muscle strength of college students with CNP is weaker than that of healthy college students in 3 different positions. It can be inferred that if the individual has too much muscle strength loss during the neck extensor muscle strength test, it can be regarded as an early warning of imminent neck pain, and it is necessary to strengthen and maintain the movement and stability of the cervical spine as soon as possible.

### 4.3. Static EMG characteristics of neck muscle in college students with CNP

Surface EMG is a technique for placing electrodes on the skin of the body surface to record bioelectrical signals in muscle activity.^[49]^ The EMG signal can reflect the fluctuation of the muscle activity function and level in a certain range. It is noninvasive, sensitive, easily accepted by patients, and has a wide application prospect.^[50]^ In recent years, surface EMG has been considered as an index to evaluate the function of muscle fatigue.^[50]^ In recent years, surface EMG has been considered as an index to evaluate the function of muscle fatigue,^[51]^ which has attracted the attention of researchers in rehabilitation medicine.^[52,53]^

The degree of fatigue induced in a muscle over a specific duration, as well as the total number of motor unit discharges, can be assessed through changes in the iEMG signal. An increase in the iEMG is indicative of some degree of muscle fatigue. When a muscle is overexerted, the EMG signal exhibits significant fluctuations.

With the continuous advancement of science and technology in modern society, EMG technology is increasingly utilized in sports and clinical medicine. EMG has good sensitivity in the prediction and diagnosis of neck pain and CNP. Because the muscles evaluated by EMG are different, it is considered the best means to assess muscle strength. In the study, it was found that the muscle tension of patients with CNP was too high, the myoelectric activity was too long, and the muscle strength was unbalanced. Ma studied the correlation between EMG changes and neck and shoulder pain dysfunction. When testing the maximum isometric muscle strength of the neck and shoulder, the surface EMG technology was used to process the EMG signal, and the amplitude probability distribution function of the maximum isometric muscle strength was compared and analyzed. In surface EMG, the average frequency is often used to reflect the degree of muscle fatigue. In contrast, the time domain index can be used as a value to evaluate muscle strength. The larger the value, the stronger the muscle strength.^[54]^

In this study, we assessed the static sEMG of the splenius capitis muscles and upper trapezius muscles using maximum isometric muscle strength. Mean and iEMG measurements were recorded for the neck muscles across 3 different positions. The results indicated that college students with CNP exhibited lower mean and iEMG values compared to their healthy counterparts.

### 4.4. Dynamic EMG characteristics of neck muscle in college students with CNP

The abnormal changes in neuromuscular activity of the head and neck in college students with CNP were studied using surface EMG. The neck muscle activity of college students with CNP was insufficient, and they lacked a flexion–relaxation response under a static state. Neck pain can lead to abnormal movement, changes in neck muscle strength, and altered neuromuscular function. EMG was used to measure the EMG of the upper trapezius muscle and the trapezius muscle during flexion and extension. The fatigue EMG signal was processed by software, and the average EMG during flexion and extension was analyzed. The surface EMG index FER was calculated using the following formula: there was no significant difference in fatigue EMG between the 2 groups. There was a significant difference between the surface EMG index flexion–extension ratio and the average EMG during flexion and extension. The average EMG of the head clip muscle increased during flexion and decreased during extension. Neck movement is controlled by complex neuromuscular systems that involve both active and passive units. During the flexion of the neck, the myoelectric activity of the neck extensor decreased (flexion–relaxation phenomenon).^[55–57]^ The maximum flexion angle of the neck increased significantly after continuous flexion. The beginning of flexion relaxation was significantly delayed during the flexion process, but the stopping angle remained unchanged. After maintaining flexion, the myoelectric activity of the cervical erector spinae increased significantly, especially in female subjects, and the FER also decreased significantly.

Surface EMG was used to observe flexion relaxation and neck muscle activity, revealing the risk of neck disease in college students. The results showed that the sternocleidomastoid muscle in the weekly pain group was more prone to fatigue than the other groups, as indicated by the average EMG. Patients with neck pain were less likely to have flexion relaxation. The FER of the trapezius muscle in the control group was lower than that in other groups and other muscles. The flexion–relaxation ratio values of the sternocleidomastoid muscle on both sides of the control group were significantly different when the pain frequency was twice a week.

In this experiment, the dynamic surface EMG of the splenius capitis and the trapezius muscle was measured during neck flexion and extension, and the average EMG ratio FER was calculated during flexion and extension. FER can reflect the coordination of flexion and extension. The comparison between college students with CNP and healthy college students showed that the flexion and extension of surface EMG indexes were relatively high, with a very significant difference (*P* < .01). When surface EMG was used to measure healthy college students, the EMG changes during active activity during flexion and relaxation were not obvious. However, in the case of complete neck flexion, the cervical extensor relaxation stage was significantly reduced, indicating flexion relaxation. The results showed that college students with CNP were significantly weaker than healthy college students in neck extensor flexion relaxation.

### 4.5. CNP college students’ splenius capitis and trapezius muscle dysfunction

Integrated EMG is the total amount of motor unit discharge generated by muscle activity in a specific time period. That is, the size of integrated EMG can simultaneously reflect the number of motor units involved in muscle activity and the size of discharge, which is positively correlated with muscle strength.^[56,58]^ The sEMG test of the neck extensor muscles included average EMG and median frequency slope. After treatment, the AEMG of the cervical erector spinae and trapezius muscles was significantly improved, and the median frequency slope of both groups of muscles was significantly reduced. Therefore, exercise therapy can effectively improve the neck muscle function of patients.^[59]^ According to the treatment methods, they were divided into an observation group and a control group. The control group received routine rehabilitation treatment. The observation group received neck muscle isometric contraction training, and the changes in neck function after intervention were compared between the 2 groups. The results showed that the neck function of both groups improved after the intervention, and the neck function of the observation group was better than that of the control group. Neck muscle isometric contraction training can significantly improve motor function in the neck, effectively relieve pain, and enhance daily living ability.^[60]^

The neck muscles are categorized into deep and superficial layers, each of which must work synergistically to facilitate movement, relaxation, and stabilization of the cervical spine. In maintaining neck position and posture, the weaker muscles are required to sustain isometric contractions over extended periods. Active muscle groups are particularly prone to fatigue during neck movements in various directions, with the splenius capitis muscles being especially susceptible to fatigue during prolonged neck flexion. College students with neck pain are more likely to experience muscle fatigue in the neck, regardless of the duration of continuous activity or pain intensity, compared to their healthy counterparts. Decreased muscle endurance and neuromuscular efficiency are considered the primary causes of CNP.^[52]^ To determine the effect of using electronic products on neck muscle function, 2 mobile phones and tablets with varying screen sizes were used while participants were seated. In contrast, the electronic products were either handheld or placed on a desktop. Three different operations (reading, typing, and playing) lasted for 90 s. The results showed that the smaller the screen of electronic products, the higher the activation of the trapezius muscle; however, there was no statistically significant difference. During neck flexion, the activation of the trapezius muscles on the left and right sides was different. Different sizes of electronic products, varying methods of use, and diverse operational methods have had a distinct impact on neck muscle activity. In a specific sitting position, the trapezius muscle was significantly activated by typing, and the activation degree of the left and right trapezius was different. In the long run, it will lead to decreased muscle function and posture asymmetry in the head and neck, and may increase the risk of neck disease.^[61,62]^

When the head of the 2 groups of CNP college students and healthy college students was in a neutral position, the contribution rate of the integral EMG of the neck muscle was 51.5% and 53.9% (splenius capitis), 48.4% and 45.3% (trapezius muscle), respectively. When the head was tilted forward naturally, the contribution rates of integral EMG to the isometric contraction of the neck muscle were 49.1% and 54.0% (splenius capitis), 50.8%, and 45.9% (trapezius muscle), respectively. When the head was in the maximum flexion position, the contribution rates of integral EMG to the isometric contraction of the neck muscle were 47.7% and 52.6% (splenius capitis), 52.1% and 47.3% (trapezius muscle), respectively. It shows that the integral EMG of the head clip muscle of college students with CNP decreases more, and the trapezius muscle decreases relatively less. Therefore, the muscle strength of the splenius capitis decreased more than that of the healthy people, and the decrease of the trapezius muscle was relatively less. It is proved that the muscle strength of the splenius capitis decreases more, and the results of the integral EMG of the neck muscle show that there are very significant differences in the head neutral position, the head natural forward position, and the head maximum flexion position. The comprehensive muscle strength and EMG indicators indicate that the muscle recruitment ability of CNP college students becomes worse when the neck muscles fight against resistance contraction in this posture.

### 4.6. Limitations and future research direction

Although this study systematically explored head and shoulder posture and cervical muscle EMG characteristics in college students with CNP, it has several limitations. First, the sample was limited to university students, which restricts the generalizability and representativeness of the findings. Second, as a cross-sectional study, it cannot establish causal relationships and does not assess the effects of any interventions. Future research should expand the sample population, adopt longitudinal or intervention-based designs, integrate psychological and lifestyle factors, and utilize digital technologies to develop early screening and intervention models for CNP, thereby enhancing the clinical value and practical application of the research.

## 5. Conclusions

College students with CNP show significant abnormalities in head–shoulder posture, decreased neck extensor strength, and altered EMG activity, including a diminished flexion–relaxation response. These findings highlight the critical role of postural and neuromuscular dysfunction in CNP, providing a valuable reference for clinical diagnosis and rehabilitation in the college student population.

## Acknowledgments

The authors wish to thank the participants for their contribution to this study.

## Author contributions

**Conceptualization:** Yanqing Yan, Jifeng Dong, Taiping Li.

**Data curation:** Yanqing Yan, Jifeng Dong, Taiping Li.

**Formal analysis:** Yanqing Yan, Jifeng Dong, Taiping Li.

**Funding acquisition:** Yanqing Yan, Taiping Li.

**Investigation:** Yanqing Yan, Jifeng Dong, Taiping Li.

**Methodology:** Yanqing Yan, Jifeng Dong, Taiping Li.

**Project administration:** Yanqing Yan, Jifeng Dong, Taiping Li.

**Resources:** Yanqing Yan, Jifeng Dong, Taiping Li.

**Software:** Yanqing Yan, Jifeng Dong, Taiping Li.

**Supervision:** Yanqing Yan, Jifeng Dong, Taiping Li.

**Validation:** Yanqing Yan, Jifeng Dong, Taiping Li.

**Visualization:** Yanqing Yan, Jifeng Dong, Taiping Li.

**Writing – original draft:** Yanqing Yan, Jifeng Dong, Taiping Li.

**Writing – review & editing:** Yanqing Yan, Jifeng Dong, Taiping Li.

## References

[1] Noormohammadpour P Mansournia MA Koohpayehzadeh J. Prevalence of chronic neck pain, low back pain, and knee pain and their related factors in community-dwelling adults in Iran: a population-based national study. Clin J Pain. 2017;33:181–7. 27258995 10.1097/AJP.0000000000000396

[2] Kahlaee AH Ghamkhar L Arab AM. The association between neck pain and pulmonary function: a systematic review. Am J Phys Med Rehabil. 2017;96:203–10. 27610549 10.1097/PHM.0000000000000608

[3] Hsu W-L Chen CP Nikkhoo M. Fatigue changes neck muscle control and deteriorates postural stability during arm movement perturbations in patients with chronic neck pain. Spine J. 2020;20:530–7. 31672689 10.1016/j.spinee.2019.10.016

[4] Chen Y Yang C Nie K Huang J Qu Y Wang T. Effects of scapular treatment on chronic neck pain: a systematic review and meta-analysis of randomized controlled trials. BMC Musculoskelet Disord. 2024;25:252. 38561733 10.1186/s12891-024-07220-8 PMC10983729

[5] Lin LH Lin T-Y Chang K-V Wu W-T Özçakar L. Pain neuroscience education for reducing pain and kinesiophobia in patients with chronic neck pain: a systematic review and meta‐analysis of randomized controlled trials. Eur J Pain. 2024;28:231–43. 37694895 10.1002/ejp.2182

[6] Maroufi N Ahmadi A Mousavi Khatir SR. A comparative investigation of flexion relaxation phenomenon in healthy and chronic neck pain subjects. Eur Spine J. 2013;22:162–8. 23053754 10.1007/s00586-012-2517-3 PMC3540320

[7] Kanchanomai S Janwantanakul P Pensri P Jiamjarasrangsi W. Risk factors for the onset and persistence of neck pain in undergraduate students: 1-year prospective cohort study. BMC Public Health. 2011;11:1–8. 21756362 10.1186/1471-2458-11-566 PMC3150268

[8] Gao Y Chen Z Chen S Wang S Lin J. Risk factors for neck pain in college students: a systematic review and meta-analysis. BMC Public Health. 2023;23:1502. 37553622 10.1186/s12889-023-16212-7 PMC10408143

[9] Chan LLY Wong AYL Wang MH Cheung K Samartzis D. The prevalence of neck pain and associated risk factors among undergraduate students: a large-scale cross-sectional study. Int J Ind Ergon. 2020;76:102934.

[10] Elvan A Cevik S Vatansever K Erak I. The association between mobile phone usage duration, neck muscle endurance, and neck pain among university students. Sci Rep. 2024;14:20116. 39209955 10.1038/s41598-024-71153-4 PMC11362573

[11] Kim SD. Effects of yogic exercise on nonspecific neck pain in university students. Complement Ther Clin Pract. 2018;31:338–42. 29066175 10.1016/j.ctcp.2017.10.003

[12] Zheng D-D Li D Cheng J-X Jin R-H. The prevalence of neck pain among online learning students: an observational study. Medicine (Baltimore). 2024;103:e39264. 39121309 10.1097/MD.0000000000039264 PMC11315509

[13] Mehri A Letafatkar A Khosrokiani Z. Effects of corrective exercises on posture, pain, and muscle activation of patients with chronic neck pain exposed to anterior-posterior perturbation. J Manipulative Physiol Ther. 2020;43:311–24. 32723668 10.1016/j.jmpt.2018.11.032

[14] Mostafaee N HasanNia F Negahban H Pirayeh N. Evaluating differences between participants with various forward head posture with and without postural neck pain using craniovertebral angle and forward shoulder angle. J Manipulative Physiol Ther. 2022;45:179–87. 35902274 10.1016/j.jmpt.2022.06.007

[15] Mahmoud NF Hassan KA Abdelmajeed SF Moustafa IM Silva AG. The relationship between forward head posture and neck pain: a systematic review and meta-analysis. Curr Rev Musculoskelet Med. 2019;12:562–77. 31773477 10.1007/s12178-019-09594-y PMC6942109

[16] Ghamkhar L Kahlaee AH. Is forward head posture relevant to cervical muscles performance and neck pain? A case–control study. Braz J Phys Ther. 2019;23:346–54. 30145129 10.1016/j.bjpt.2018.08.007 PMC6630105

[17] Venie X Guoqiang F Yuan Z. Study on massage rehabilitation technique of cervical spondylotic radiculopathy. Arch Clin Psychiatry. 2022;49:6.

[18] Liu X Guo Z. Clinical effect of yoga in treating cervical spondylosis. Investig Clín. 2020;61:982–9.

[19] Ng D McNee C Kieser J Farella M. Neck and shoulder muscle activity during standardized work-related postural tasks. Appl Ergon. 2014;45:556–63. 23972454 10.1016/j.apergo.2013.07.012

[20] Gadotti I Bérzin F Biasotto-Gonzalez D. Preliminary rapport on head posture and muscle activity in subjects with class I and II. J Oral Rehabil. 2005;32:794–9. 16202042 10.1111/j.1365-2842.2005.01508.x

[21] Bruno Garza JL Eijckelhof BHW Huysmans MA. Prediction of trapezius muscle activity and shoulder, head, neck, and torso postures during computer use: results of a field study. BMC Musculoskelet Disord. 2014;15:1–14. 25186007 10.1186/1471-2474-15-292 PMC4161866

[22] Murray M Lange B Chreiteh SS. Neck and shoulder muscle activity and posture among helicopter pilots and crew-members during military helicopter flight. J Electromyogr Kinesiol. 2016;27:10–7. 26852114 10.1016/j.jelekin.2015.12.009

[23] Fernández-de-Las-Peñas C Cuadrado M Pareja J. Myofascial trigger points, neck mobility and forward head posture in unilateral migraine. Cephalalgia. 2006;26:1061–70. 16919056 10.1111/j.1468-2982.2006.01162.x

[24] Sheikhhoseini R Shahrbanian S Sayyadi P O'Sullivan K. Effectiveness of therapeutic exercise on forward head posture: a systematic review and meta-analysis. J Manipulative Physiol Ther. 2018;41:530–9. 30107937 10.1016/j.jmpt.2018.02.002

[25] Vikne H Bakke ES Liestøl K Engen SR Vøllestad N. Muscle activity and head kinematics in unconstrained movements in subjects with chronic neck pain; cervical motor dysfunction or low exertion motor output? BMC Musculoskelet Disord. 2013;14:1–12. 24188070 10.1186/1471-2474-14-314 PMC3840692

[26] Johnston V Jull G Souvlis T Jimmieson NL. Neck movement and muscle activity characteristics in female office workers with neck pain. Spine. 2008;33:555–63. 18317202 10.1097/BRS.0b013e3181657d0d

[27] Woodhouse A Vasseljen O. Altered motor control patterns in whiplash and chronic neck pain. BMC Musculoskelet Disord. 2008;9:1–10. 18570647 10.1186/1471-2474-9-90 PMC2446396

[28] Cheng Z-J Zhang S-P Gu Y-J. Effectiveness of Tuina therapy combined with Yijinjing exercise in the treatment of nonspecific chronic neck pain: a randomized clinical trial. JAMA Netw Open. 2022;5:e2246538–e2246538. 36512354 10.1001/jamanetworkopen.2022.46538 PMC9856335

[29] Treede R-D Rief W Barke A. A classification of chronic pain for ICD-11. Pain. 2015;156:1003–7. 25844555 10.1097/j.pain.0000000000000160 PMC4450869

[30] Blanpied PR Gross AR Elliott JM. Neck pain: revision 2017: clinical practice guidelines linked to the international classification of functioning, disability and health from the orthopaedic section of the American Physical Therapy Association. J Orthop Sports Phys Ther. 2017;47:A1–A83.

[31] Shaghayegh Fard B Ahmadi A Maroufi N Sarrafzadeh J. Evaluation of forward head posture in sitting and standing positions. Eur Spine J. 2016;25:3577–82. 26476717 10.1007/s00586-015-4254-x

[32] Watson DH Trott PH. Cervical headache: an investigation of natural head posture and upper cervical flexor muscle performance. Cephalalgia. 1993;13:272–84; discussion 232. 8374943 10.1046/j.1468-2982.1993.1304272.x

[33] Han J-T Lee J-H Yoon C-H. The mechanical effect of kinesiology tape on rounded shoulder posture in seated male workers: a single-blinded randomized controlled pilot study. Physiother Theory Pract. 2015;31:120–5. 25264014 10.3109/09593985.2014.960054

[34] Kazeminasab S Nejadghaderi SA Amiri P. Neck pain: global epidemiology, trends and risk factors. BMC Musculoskelet Disord. 2022;23:1–13. 34980079 10.1186/s12891-021-04957-4 PMC8725362

[35] Warda DG Nwakibu U Nourbakhsh A. Neck and upper extremity musculoskeletal symptoms secondary to maladaptive postures caused by cell phones and backpacks in school-aged children and adolescents. Healthcare (Basel). 2023;11:819. 36981476 10.3390/healthcare11060819 PMC10048647

[36] Dere T Alemdaroğlu-Gürbüz I. Muscular endurance and its association with neck pain, disability, neck awareness, and kinesiophobia in patients with chronic neck pain. Somatosens Mot Res. 2024;41:134–41. 36897182 10.1080/08990220.2023.2186390

[37] Hasan S Iqbal A Alghadir AH Alonazi A Alyahya D. The combined effect of the trapezius muscle strengthening and pectoralis minor muscle stretching on correcting the rounded shoulder posture and shoulder flexion range of motion among young Saudi females: a randomized comparative study. Healthcare (Basel). 2023;11:500. 36833034 10.3390/healthcare11040500 PMC9956189

[38] Guduru RKR Domeika A Domeikienė A. Effect of rounded and hunched shoulder postures on myotonometric measurements of upper body muscles in sedentary workers. Appl Sci. 2022;12:3333.

[39] Thigpen CA Padua DA Michener LA. Head and shoulder posture affect scapular mechanics and muscle activity in overhead tasks. J Electromyogr Kinesiol. 2010;20:701–9. 20097090 10.1016/j.jelekin.2009.12.003

[40] Ertekin E Günaydin OE. Neck pain in rounded shoulder posture: clinico‐radiologic correlation by shear wave elastography. Int J Clin Pract. 2021;75:e14240. 33971068 10.1111/ijcp.14240

[41] Nitayarak H Charntaraviroj P. Effects of scapular stabilization exercises on posture and muscle imbalances in women with upper crossed syndrome: a randomized controlled trial. J Back Musculoskelet Rehabil. 2021;34:1031–40. 34151819 10.3233/BMR-200088

[42] Wannaprom N Jull G Treleaven J Warner MB Uthaikhup S. Axioscapular and neck extensor muscle behavior during isometric shoulder exertions in patients with nonspecific neck pain with and without a scapular downward rotation posture. Gait Posture. 2023;101:41–7. 36724655 10.1016/j.gaitpost.2023.01.010

[43] Requejo-Salinas N Fernández-Matías R Cadogan A. Neck or shoulder? Establishing consensus for spine screening in patients with shoulder pain: an international modified Delphi study. Phys Ther. 2024;105:pzae133. 10.1093/ptj/pzae133 39239842

[44] Choi W. Effect of 4 weeks of cervical deep muscle flexion exercise on headache and sleep disorder in patients with tension headache and forward head posture. Int J Environ Res Public Health. 2021;18:3410. 33806089 10.3390/ijerph18073410 PMC8037445

[45] Multanen J Häkkinen A Kautiainen H Ylinen J. Associations of neck muscle strength and cervical spine mobility with future neck pain and disability: a prospective 16-year study. BMC Musculoskelet Disord. 2021;22:1–10. 34715847 10.1186/s12891-021-04807-3 PMC8556991

[46] Colman D Demoulin C Vanderthommen M. Exercise therapy including the cervical extensor muscles in individuals with neck pain: a systematic review. Clin Rehabil. 2023;37:1579–610. 37424506 10.1177/02692155231184973

[47] Jull GA Falla D Vicenzino B Hodges PW. The effect of therapeutic exercise on activation of the deep cervical flexor muscles in people with chronic neck pain. Man Ther. 2009;14:696–701. 19632880 10.1016/j.math.2009.05.004

[48] Reddy RS Tedla JS Dixit S Abohashrh M. Cervical proprioception and its relationship with neck pain intensity in subjects with cervical spondylosis. BMC Musculoskelet Disord. 2019;20:1–7. 31615495 10.1186/s12891-019-2846-z PMC6794723

[49] Perpetuini D Formenti D Cardone D. Can data-driven supervised machine learning approaches applied to infrared thermal imaging data estimate muscular activity and fatigue? Sensors (Basel). 2023;23:832. 36679631 10.3390/s23020832 PMC9863897

[50] Kim S-Y Koo S-J. Effect of duration of smartphone use on muscle fatigue and pain caused by forward head posture in adults. J Phys Ther Sci. 2016;28:1669–72. 27390391 10.1589/jpts.28.1669 PMC4932032

[51] Liu Y. Muscle fatigue judgement based on neck electromyogram signals. MEDS Clin Med. 2023;4:43–51.

[52] Yan Z-W Yang Z Zhao F-L. Effect of sling exercise therapy on surface electromyography and muscle thickness of superficial cervical muscle groups in female patients with chronic neck pain. J Back Musculoskelet Rehabil. 2023;36:387–97. 36278336 10.3233/BMR-220030

[53] Nobe R Yajima H Takayama M Takakura N. Characteristics of surface electromyograph activity of cervical extensors and flexors in nonspecific neck pain patients: a cross-sectional study. Medicina (Kaunas). 2022;58:1770. 36556971 10.3390/medicina58121770 PMC9781307

[54] Pires PF Packer AC Dibai-Filho AV Rodrigues-Bigaton D. Immediate and short-term effects of upper thoracic manipulation on myoelectric activity of sternocleidomastoid muscles in young women with chronic neck pain: a randomized blind clinical trial. J Manipulative Physiol Ther. 2015;38:555–63. 26387859 10.1016/j.jmpt.2015.06.016

[55] Shamsi H Khademi-Kalantari K Akbarzadeh-Baghban A Izadi N Okhovatian F. Cervical flexion relaxation phenomenon in patients with and without non-specific chronic neck pain. J Back Musculoskelet Rehabil. 2021;34:461–8. 33492275 10.3233/BMR-200137

[56] Wang D Ding Y Wu B. Cervical extensor muscles play the role on malalignment of cervical spine: a case control study with surface electromyography assessment. Spine. 2021;46:E73–9. 33038198 10.1097/BRS.0000000000003742

[57] He P Yang Y Wang M. Is the disappearance of the cervical flexion–relaxation phenomenon associated with cervical degeneration in healthy people? Eur Spine J. 2024;33:2997–3007. 38869650 10.1007/s00586-024-08355-x

[58] Radecka A Lubkowska A. The usefulness of surface electromyography in rehabilitation and physiotherapy: systematic review. Pomeranian J Life Sci. 2020;66:49–56.

[59] Tsang SMH Chan KT Ho PL Kwok JC Tse DH Tsoi HH. Comparison between velocity‐specific exercise and isometric exercise on neck muscle functions and performance: a randomised clinical trial. BMC Musculoskelet Disord. 2021;22:81. 33446159 10.1186/s12891-021-03943-0 PMC7809848

[60] Wu B Yuan H Geng D Zhang L Zhang C. The impact of a stabilization exercise on neck pain: a systematic review and meta-analysis. J Neurol Surg A Cent Eur Neurosurg. 2020;81:342–7. 32143228 10.1055/s-0039-3400953

[61] Chen Y-L Chan Y-C Alexander H. Gender differences in neck muscle activity during near-maximum forward head flexion while using smartphones with varied postures. Sci Rep. 2024;14:12994. 38844574 10.1038/s41598-024-63734-0 PMC11156881

[62] Song D Park D Kim E Shin G. Neck muscle fatigue due to sustained neck flexion during smartphone use. Int J Ind Ergon. 2024;100:103554.

